# Characterization and Ofloxacin Adsorption Studies of Chemically Modified Activated Carbon from Cassava Stem

**DOI:** 10.3390/ma15155117

**Published:** 2022-07-22

**Authors:** Nurul Syuhada Sulaiman, Mohd Hazim Mohamad Amini, Mohammed Danish, Othman Sulaiman, Rokiah Hashim, Samet Demirel, Gaye Kose Demirel

**Affiliations:** 1 Division of Bioresource Technology, School of Industrial Technology, Universiti Sains Malaysia, Gelugor 11800, Malaysia; nurulsyuhada8496@gmail.com (N.S.S.); danish@usm.my (M.D.); osulaiman@gmail.com (O.S.); hrokiah@gmail.com (R.H.); 2 Faculty of Bioengineering and Technology, University Malaysia Kelantan, Jeli Campus, Jeli 17600, Malaysia; 3 Faculty of Forestry, Karadeniz Technical University, Trabzon 61080, Turkey; sdemirel@ktu.edu.tr (S.D.); gkose@hotmail.com (G.K.D.)

**Keywords:** cassava, ofloxacin, adsorption, kinetic study, activated carbon

## Abstract

Cassava is a type of crop popular in Asian countries. It can be easily cultivated and grows to a mature plant in 9 months. Considering its availability, this work studied activated carbon based on cassava stem. Ofloxacin was chosen as the adsorbate, simulating the wastewater from the pharmaceutical industry. Cassava stem was ground into particles and heated to the activated state, 787 °C. The cassava-stem-activated carbon was further treated with the surface modifier, namely sodium hydroxide and zinc chloride, to study the improvement in ofloxacin adsorption. Prepared adsorbents were characterised using the SEM, FT-IR, XRD, DSC and TGA methods before being evaluated through batch adsorption, thermodynamic, and kinetic studies. The surface area analysis indicates that treatment of the activated carbon with NaOH and ZnCl_2_ increases the surface area due to the removal of organic content by the chemicals. Better ofloxacin adsorption of all activated carbon samples can be obtained with solutions at pH 8. An endothermic reaction was predicted, shown by higher ofloxacin adsorption at a higher temperature, supported by a positive value of ΔH° in the thermodynamic studies. The negative values of ΔG° revealed that adsorptions were spontaneous. The higher R^2^ values indicate that the adsorption process follows the pseudo-second-order equation of kinetic study. The maximum adsorption capacities are 42.37, 62.11, 62.89 and 58.82 mg/g for raw cassava stem (RC), cassava-stem-activated carbon (AC), NaOH-modified cassava-stem-activated carbon (NAC), and ZnCl_2_ modified cassava-stem-activated carbon (ZAC). The adsorption capacity is good compared to previous works by other researchers, making it a possible alternative material for the pharmaceutical industry’s wastewater treatment.

## 1. Introduction

Ofloxacin is an antibiotic from the fluoroquinolone chemical group, widely used to treat bacterial infections in healthcare sectors [1]. The human body cannot decompose ofloxacin. Therefore, it is removed through urination [2]. Continuous input of ofloxacin into the aquatic environment increases the potential risk to marine organisms and ecosystem equilibrium. It was found that the presence of ofloxacin in surface water negatively affects cyanobacteria and aquatic plants [3]. Various studies on the existence of ofloxacin found up to 17.7 μg/L of the antibiotic in freshwater sources [3,4].

To clean the wastewater from contaminants, many techniques have been developed, including chemical precipitation, ion exchange, coagulation and flocculation, complexation, cementation, membrane filtration and adsorption [5]. Among all, the adsorption technique is the process applied the most. The adsorption process is simple, convenient and effortless in operation and adsorbent production. Various adsorbent materials have been researched for ofloxacin clearance from water, as shown in Table 1.

Activated carbon is a versatile material, with usage ranging from water treatment [16] to supercapacitors [17] and dye-sensitized solar cells [18]. The adsorption process using activated carbon is commonly applied to clear water contaminants. Common contaminants that could be removed by activated carbon include dyes and heavy metals, such as methylene blue [19], methyl orange [20], malachite green [21], cadmium [22], plumbum [23] and zinc [24]. Removal of the cyanotoxin in cylindrospermopsin form [25], oligoheterocyclic waste molecules for photonic generation of electricity, tetracycline from agricultural residue [26] and Metronidazole antibiotics [27] from waste water are some recently studied activated carbon applications. Despite the higher cost of activated carbon production [22], its usage as a contaminant adsorbent is still popular due to its practicality. Compared to other remedial techniques, the adsorption process was chosen due to the lower operational cost, easier application and reduced waste [28]. Finding a low-cost precursor for activated carbon production is a must, most probably from waste materials. Cassava biomass waste is one of the potential precursors. Cassava is easy to grow and has drought-tolerant ability [29]. It is an essential crop in tropical countries where its usage is not only limited for food, but also covers animal feed, green energy and raw material in industrial sectors [30]. It is estimated that 8 million farmers are cultivating cassava (*Manihot esculanta*) in Asia alone [31]. 

Cassava was planted mainly for its tuber, which contains a carbohydrate source. The cassava stems can grow up to 5 m in height with diameters ranging from 2.5 to 8.0 cm. After harvest, only 10–20% of the stems are spared for replanting, while the others are abandoned to rot. For every kilogram of the tuber root production, half of the mass comes from the stem part [32]. The cassava tuber root can be harvested from 7 months to 24 months [33]. This short planting cycle produces a lot of biomass waste that can be turned into something more beneficial. 

This work investigates the removal of ofloxacin from wastewater using activated carbon produced using the cassava stem. The activated carbon produced was further surface treated with Sodium Hydroxide and Zinc Chloride to increase ofloxacin uptake percentage. Chemical oxidation using chemicals such as the NaOH and Zinc chloride on the carbon surface added more oxygen functional groups. The electronegative polarity of the oxygen functional groups increases the ability to adsorb both aqueous heavy metal compounds and cations [34]. The prepared activated carbon was characterised and evaluated for its adsorption ability by using batch adsorption, thermodynamic and kinetic studies. Few studies have been performed on surface-modified activated carbon for ofloxacin adsorption. Therefore, this work could fill the knowledge gap by investigating the new potential use of cassava-stem-based activated carbon. 

## 2. Materials and Methods

### 2.1. Material Acquisition

Six-month-old samples of cassava stem were freshly cut from the living tree of a local farmer in Raub, Pahang, Malaysia. The samples were taken approximately 5 cm from the bottom and 15 cm from the top. The inner soft-core part was separated from the stem, and the sample was dried in a convection oven at 105± °C to achieve moisture content under 10% for further processing. Dried cassava stem samples were crushed using a grinder and sieved to obtain particles between 500 μm to 1 mm in size range after sieving [35]. The sieved particles were stored in a closed container for further experiment. 

### 2.2. Adsorbent and Adsorbate Preparation

After determination of the cassava stem particle moisture content, the sample was placed into a graphite reactor with a closed lid and heated using an electrical furnace (RISEN PID-96T) at the temperature of 787 °C, as obtained from RSM analysis in our previous work [32]. The temperature was maintained for 146 min, then left to cool before the activated carbon was collected and stored for further use. The surface modification of the activated carbon samples was carried out using 2.0 M sodium hydroxide (NaOH) and zinc chloride (ZnCl_2_) as the dehydrating agent. Previous research found their enhancing effect on many carbon-based lignocellulose materials [36]. Approximately 5 g of the activated carbon samples were mixed with 50 mL of the dehydrating agents. The mixture was then heated for 2 h at a temperature of 100 °C before repeated washing with distilled water to ensure a stable pH value. The samples were then dried overnight in an oven at 50 °C. The NaOH-treated activated carbon was code-named NAC, ZnCl_2_-treated activated carbon was named ZAC, raw activated carbon was named AC and the raw cassava stem was named RC.

Ofloxacin (OFX) as the adsorbate was purchased from Sigma-Aldrich with a CAS number of 82419-36-1. The solution of OFX with a concentration of 1000 ppm was carried out by diluting approximately 1 g of OFX powder with deionised water in 1000 mL volumetric flasks, then undergoing further serial dilutions to obtain the desired concentrations. 

### 2.3. Proximate Analysis of Carbon Samples

The standard test method ASTM D1762–84 [37] was referred to for the proximate analysis of the prepared carbon samples. Determination of moisture content, volatile matter and ash content are covered by this standard, calculated using Equations (1)–(3), respectively:

(1) $$\mathrm{Moisture}\;\mathrm{content},\;\%=\frac{A-B}{A}\times 100$$

where *A* is the initial air-dry mass of sample (g) and *B* is the mass of the sample (g) after drying at 105 °C;

(2) $$\mathrm{Volatile}\;\mathrm{matter},\;\%=\frac{B-C}{B}\times 100$$

where *C* is the mass of the samples (g) after drying at 450 °C; and

(3) $$\mathrm{Ash}\;\mathrm{content},\;\%=\frac{D}{B}\times 100$$

where *D* is the mass of the white residue (g). These results were expressed to the second decimal place.

### 2.4. pH at a Zero-Point Charge (pHzpc)

The solid addition method was employed to determine the pHzpc of each adsorbent [38]. Solutions of 0.03 M KNO_3_ were placed in conical flasks at 50 mL each. The initial pH was adjusted to a predetermined value between 1.5 and 11.5 using 0.1 M HCl or 0.1 M NaOH solutions. A 0.25 g of the prepared adsorbent was added to the solutions and left to stir for 24 h before the final pH value was measured. The value of pHzpc was determined by plotting the initial pH values versus the final pH values. The intersection of the plot with the straight line of initial pH = final pH plot was indicated as the pHzpc value.

### 2.5. Other Characterisations

Scanning electron microscopy (SEM) and energy dispersive X-ray analysis (EDX) were performed on the gold-coated samples using an acceleration of 5 kV, completed by a scanning electron microscope (Model Supra 50 VP). Surface area analysis was carried out using ASAP 2020 V3.04H (Micromeritics^®^). The sample was automatically degassed at 90 °C for 1 h for the first stage and 350 °C for 4 h for the second stage, with the N_2_ adsorption–desorption isotherm taken at −195.798 °C. The primary content of organic materials, which are carbon (C), hydrogen (H), nitrogen (N) and sulphur (S), were measured by a varioMICRO (V3.1.1) CHNS Elemental Analyser. The surface functional group of the prepared materials was determined using a Fourier transform infrared spectrometer (Nicolet Avatar 360 ESP FTIR) with 64 times scanning at a resolution of 4 cm^−1^ over a region of 4000 to 400 cm^−1^. The crystallinity index was measured using a Kristalloflex D-5000 X-ray diffraction system (Siemens, Munich, Germany). Scanning was performed at a diffraction angle 2θ ranging from 1.5° to 80°, corresponding to a scanning speed of 0.02° and 2°/min [39]. The thermal analysis was carried out under a nitrogen atmosphere with a heating rate set at 20 °C /min over temperatures ranging between 30 and 800 °C, using a Mettler Toledo TGA/SDTA851^e^ thermogravimeter (Mettler Toledo Corp., Greifensee, Switzerland). 

### 2.6. Batch Adsorption Studies

Batch adsorption studies were conducted according to [40]. Generally, the batch adsorption studies were performed by mixing 50 mL of ofloxacin as the adsorbate in a stoppered conical flask, shaken using 100 rpm speed at a predetermined temperature and for a predetermined contact time. At the end of shaking, mixtures were filtered through Whatman filter paper No. 2, and the filtered solution was analysed using a UV–Vis spectrophotometer for their final concentration. The parameters studied are tabulated in Table 2. The percentage of adsorbate adsorption by the adsorbents was computed using Equation (4):

(4) $$\mathrm{Adsorption},\;\%=\frac{\left(Ci-Ce\right)}{Ci}\times 100$$

where *Ci* and *Ce* are the initial and equilibrium concentration of ofloxacin (ppm) in the solution, respectively.

### 2.7. Isothermic, Kinetic and Thermodynamic Studies

The isothermic studies were performed at 35, 45 and 55 °C. The Langmuir and Freundlich isotherm was adopted to measure the adsorption capacity of ofloxacin onto the activated carbon [41]. Kinetic studies were carried out and analysed using the pseudo-first-order kinetic model and pseudo-second-order kinetic model, based on the work by de Franco et al. [42]. The thermodynamic study determined the thermodynamic parameters, including changes in standard enthalpy (∆H), standard entropy (∆S) and Gibbs free energy (∆*G*). ∆*H* and ∆*S* were calculated according to the Van’t Hoff equation, as given by Equation (5):

(5) $$lnKq=\frac{\Delta S}{R}-\frac{\Delta H}{RT}.\;$$

The *K_q_* value was calculated using Equation (6):

(6) $$Kq=\frac{qe\;}{Ce}.\;$$

where *q*_e_ and *C*_e_ are the equilibrium concentrations of ofloxacin on the adsorbent and in the solution, respectively. The Gibbs free energy ∆*G* was verified using Equation (7):

(7) ΔG=−RT lnKq

where *R* is the universal gas constant (8.314 JK^−1^ mol^−1^), T is the absolute temperature, and *K* is the thermodynamic equilibrium constant.

## 3. Results and Discussion

### 3.1. Proximate Analysis

The proximate analysis of both activated cassava stem and surface-modified activated cassava stem are presented in Table 3, with the raw cassava stem as a comparison. It indicates that the overall percentage of moisture content is below 10%, a significant reduction compared to the raw sample, as the result of the heating process. While the value of moisture content varies slightly between 6.83% and 6.39% for the activated carbon, it has been proven that there is no correlation between the percentages of moisture content with the adsorption power of the activated carbon [43]. The volatile content is the biomass content that was released as the biomass was heated to 400–500 °C. Raw cassava stem’s high volatile content at 84.80% dropped to 47.07–59.55% after activation. The value is considered high and useful when the carbon is used as a fuel and energy source [44].

The ash content of the cassava-based activated carbon ranged from 3.42% to 5.77%. The increment in the ash content was observed after the surface modification process. The ash contents were derived from the starting material, adding the combustible portion to the sample [45]. The residual of the modification agent was entrapped in the final product, where the ash formed reduced the hydrophobicity of the activated carbon [46]. 

The fixed carbon content of the activated cassava stem was higher than its raw content, at a maximum 37.25% difference. The activated cassava stem also showed a higher fixed carbon content when compared to the surface-modified activated cassava stem, with a difference ranging from 3.68% to 15.35%. The surface-modified activated cassava stems contain higher volatile and ash content, which decreased its fixed carbon [47]. 

### 3.2. Surface Area Analysis by Nitrogen Adsorption

Table 4 summarises all of the data obtained from the N_2_ adsorption analysis. The nonactivated raw cassava stem shows the lowest S_BET_ (0.765 m^2^/g). The NAC has the largest S_BET_ at 847.725 m^2^/g. The alkaline solution can effectively remove the organic materials within the pores of the activated cassava stem, thus resulting in the highest surface area [48]. The ZAC has a SBET value between the AC and NAC, which might be due to the zinc chloride salt introduced by the ZnCl_2_ treatment deposited in the pores of the activated cassava stem [46]. Tumirah et al. [49] also obtained a low BET surface area on their sample after exposure to a low concentration of ZnCl_2_, and recorded the presence of remaining zinc chloride salt even after a thorough washing process.

**Figure 1:**
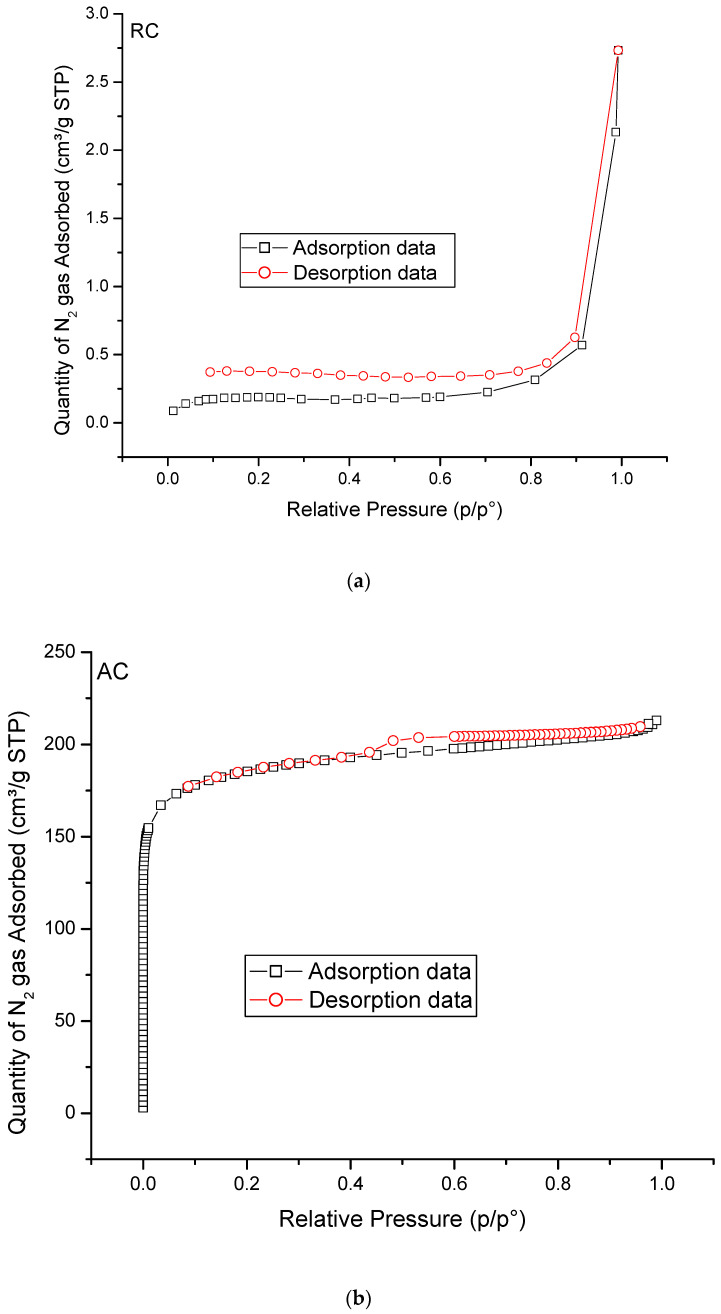

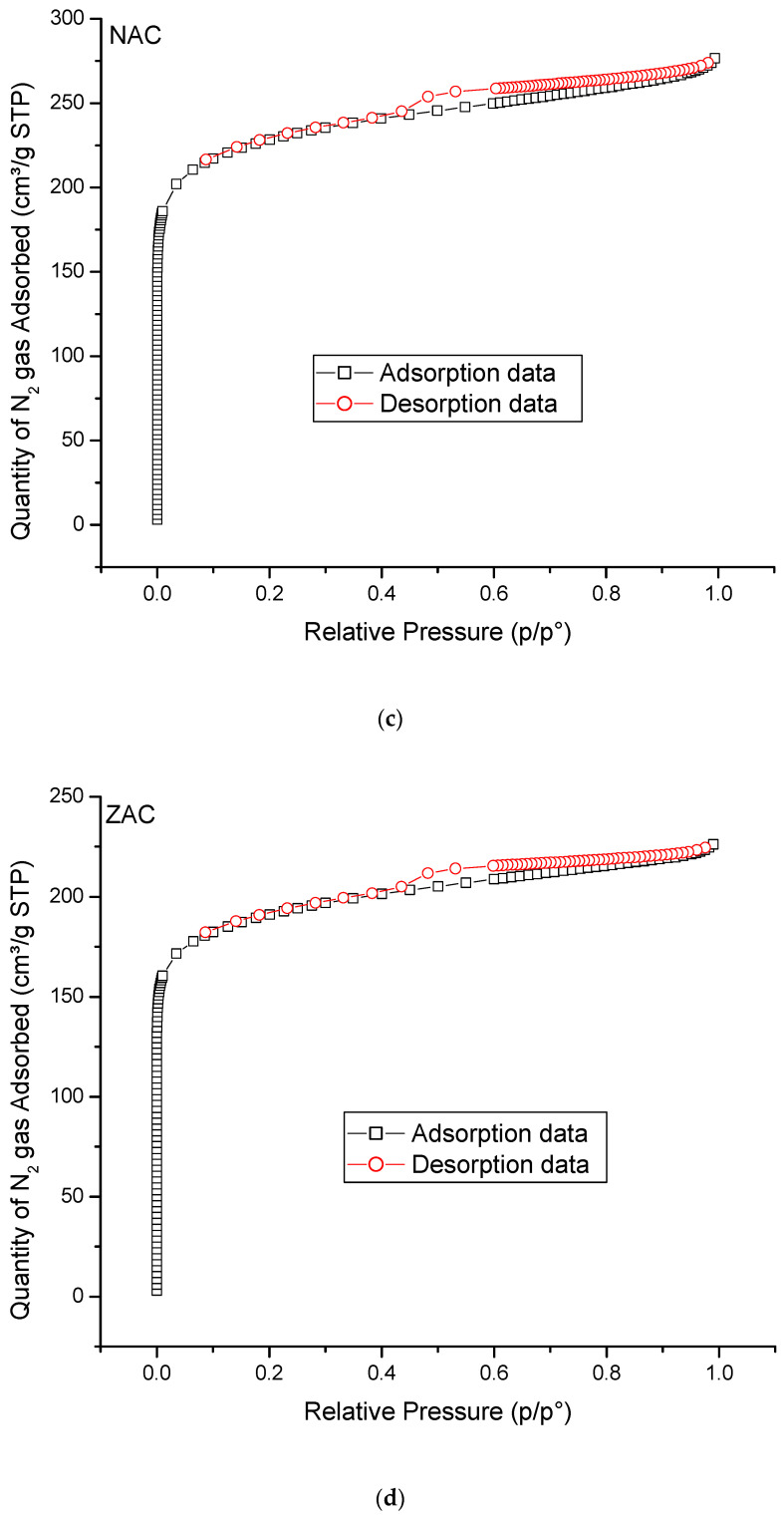

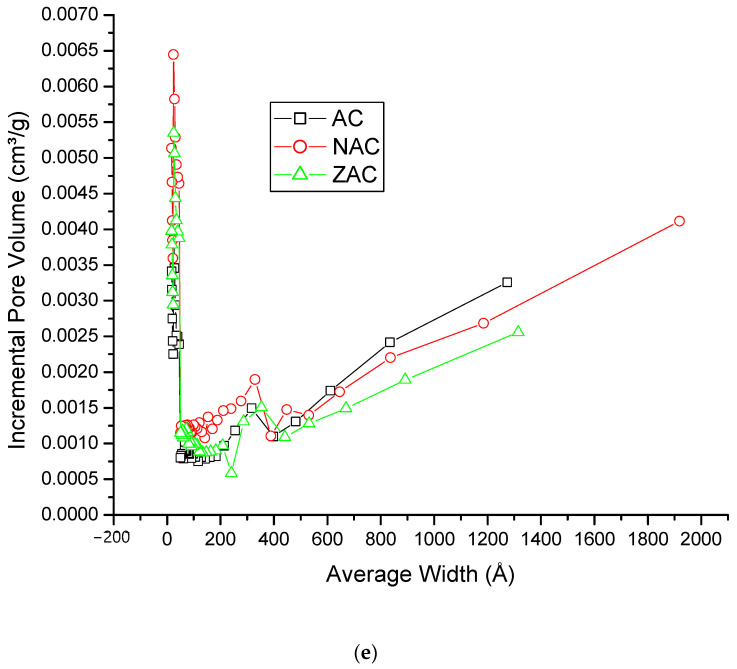
(**a**) N_2_ adsorption—desorption curves of BET surface analysis for RC. (**b**) N_2_ adsorption–desorption curves of BET surface analysis for AC. (**b**) N_2_ adsorption—desorption curves of BET surface analysis for AC. (**c**) N_2_ adsorption - desorption curves of BET surface analysis for NAC. (**d**) N_2_ adsorption—desorption curves of BET surface analysis for ZAC. (**e**) The maximum pore volume of the prepared samples.

It can be seen from Figure 1a–d that the RC adsorbents give a type II isotherm, which is the normal form of isotherm obtained with nonporous or microporous adsorbents. This type of isotherm represents unrestricted monolayer–multilayer adsorption. At the beginning of adsorption, the surface of the adsorbent achieves equilibrium, and then multilayer adsorption begins. The other adsorbents: AC, NAC, and ZAC, have a type I isotherm according to IUPAC classification of adsorption isotherms. Type I isotherms are given by microporous solids having relatively small surfaces. The pore size distribution in the adsorbents is also represented in Figure 1e; the maximum pore volumes in the adsorbents were contained within the pore size range of 1–20 nm. The average pore size for RC is too small to be detected by the analysis.

The NAC shows the largest average pore size but is not much different from the ZAC. According to the International Union of Pure and Applied Chemistry, IUPAC notation, the cassava stem pores can be categorised as micropores, which indicate pore size below 2 nm. The high holocellulose and low lignin content of cassava stem make a great precursor to produce activated carbon with microsized pores [50]. The analysis also showed that the volume of micropores (V_mic_) is larger than the volume of mesopores (V_mes_) for all samples. The micropore surface area (S_mic_) contributes more to the total S_BET_ than the mesopore surface area (S_mes_). The activation temperature used in this study was 787 °C. According to Adinata et al. [51], high numbers of micropores are produced at less than 800 °C. The macropores are first formed during the activation process, followed by the formation of mesopores as secondary channels that are created in the walls of the macropores. The attack on the planes within the raw material structure forms the micropores, and the chemical treatment on the surface increases the formation of pores on the adsorbents [49]. 

### 3.3. Scanning Electron Microscopy (SEM) and Energy Dispersive X-ray (EDX) Analysis

**Figure 2:**
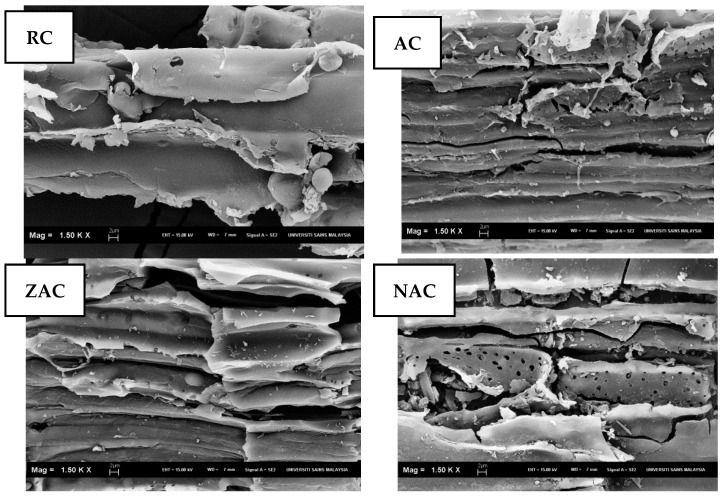
The SEM images of cassava stem adsorbent samples (1500× magnification).

The SEM examination shown in Figure 2 indicates that the raw cassava stem has a cleaner surface than other activated samples. High temperature applied during the activation process creates a lot of cracks and pores [52] due to the breakdown of the sample matrix [53]. 

The elements present on the surface of representative cassava stem adsorbents were analysed through energy dispersive X-ray (EDX) analysis, as tabulated in Table 5. All adsorbents showed a high percentage of oxygen (O), above 70%, followed by Carbon (C) at 26.26–27.19%. Potassium (K) is the only ash content. The inherent ash content elements of calcium (Ca) and magnesium (Mg) were found on the activated cassava stem (Salman 2014; Wei et al. 2014). The sodium (Na), chlorine (Cl), calcium (Ca) and zinc (Zn) elements come from the treatment chemicals, which are known as free ash [54].

The elements K, Ca and Mg that were present on the surface of the activated cassava stem were also reduced after the modification treatment due to the removal of inorganic contents by the action of ZnCl_2_ and NaOH. The high adsorption capacity was obtained through the ion exchange mechanism [55], showing that the surface-modified activated cassava stem samples might serve as suitable adsorbents than the nonmodified activated cassava stem. 

### 3.4. Elemental Analysis

Elemental analysis results are tabulated in Table 6. Carbon and oxygen are the major elements in all samples. Increment in carbon content occurs after the activation process. Volatile matter containing oxygen, hydrogen, nitrogen and sulphur were evaporated from the carbonaceous part throughout the decomposition, leading to an increase in carbon content percentage. The chemical modification of the activated cassava stem leads to a minor decrease in carbon content due to the degradation effect of those chemicals on the activated carbon. Both samples treated with different chemicals confirm this theory. 

### 3.5. Fourier Transform Infrared Spectroscopy (FTIR)

**Figure 3:**
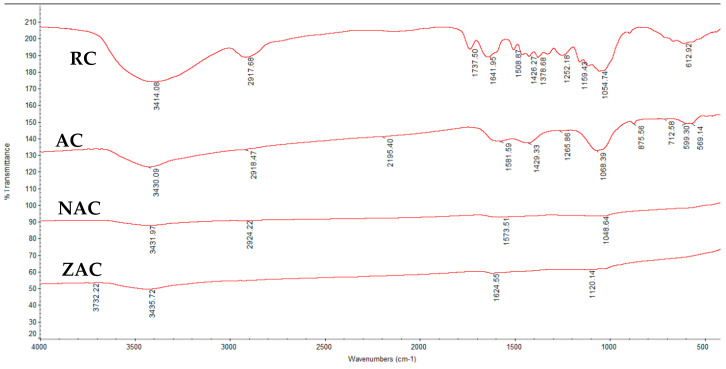
The FTIR spectra for all types of cassava stem adsorbents.

Figure 3 illustrates the FTIR spectra displaying the standard components or functional groups of lignin, cellulose and hemicellulose at wavenumbers of 4000 to 400 cm^−1^. The activation process removes many functional groups from the spectrum or shows less intensity. The thermal degradation effect destroys intermolecular bonding during the activation process [56]. 

The O-H stretching vibration in hydroxyl groups of phenols was detected at a bandwidth around 3446.18–3414.08 cm^−1^ for all samples [57]. The decrease in intensity shown in this region is attributed to the loss of oxygen during the activation and surface modification process [58]. The other identical peaks were found at bandwidths around 2926.22–2913.15 cm^−1^, which indicates the presence of C-H stretching related to alkane groups [59]. Their bending vibrations of CH_2_ were detected at 1465.92–1429.33 cm^−1^, and C-C multiple bond stretching was found at wavelengths ~2300 and ~2100 cm^−1^ [60].

The FTIR spectra of surface-modified activated cassava stem showed little difference in the surface functionality compared to raw and activated cassava stem. The Na-C exhibited C=O stretching in the quinone structure of carbonyl groups; CH_3_ of aromatic methyl groups; C-O-C stretching vibrations in ethers, esters or phenol groups; and alcohol groups (C-OH). The new peak at 606.71 cm^−1^ refers to the C=C bond of alkenes [60]. 

Additionally, the remaining peaks available on the surface of activated cassava stem modified with ZnCl_2_ are similar to the functional groups found on the surface of raw cassava stem, but with significant decreases in intensity. The presence of ester stretching of lactone, C=O stretching of carbonyl, CH_3_ of aromatic methyl groups, CHOH stretching and the alcohol groups (C-OH) were also detected in ZAC. 

### 3.6. pH at Zero Point Charge (pHzpc)

The pHzpc value determines the pH at which the adsorbent surface has net electrical neutrality [61], where the acidic or basic functional groups no longer contribute to the pH of the solution. Table 7 presents the pHzpc value for all samples, where the lowest pHzpc value was shown by raw cassava stem with its pHzpc value in a weak acidic range [62].

The alkaline pHzpc of activated cassava stem was contributed by a large amount of alkaline ash elements such as Mg and K, as shown by EDX results (Meis et al. 2010). The NAC showed a similar value, probably due to the presence of Na (alkali metal) together with the ash elements. The treatment of activated carbon with NaOH replaces the H + with Na + ion, resulting in higher basicity of the carbon [63]. 

ZAC showed a neutral pHzpc value due to the additional loss of ash elements compared to the activated cassava stem. The presence of acidic lactone detected by the FTIR analysis contributes to the decrease in the basicity of the activated cassava stem.

### 3.7. X-ray Diffractometry (XRD) Analysis

**Figure 4:**
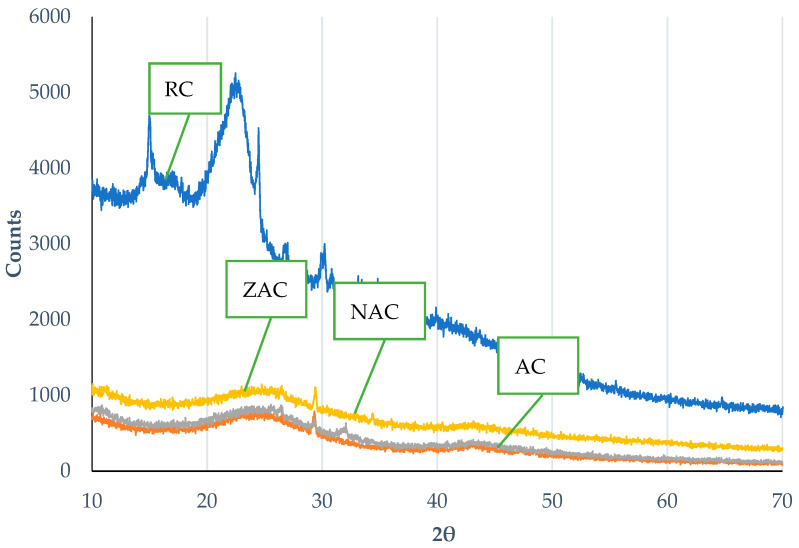
The X-ray diffractogram for adsorbent samples.

The XRD diffractogram displays the amorphous nature of all cassava stem adsorbents, as shown in Figure 4. The raw cassava stem shows the lowest CI, while activation reduced the amorphous structure by 22.2%. Surface modification has little effect on the CI. High hemicellulose and lignin contribute to a higher amorphous structure [64]. The high temperature used in the activation process degrades this structure, leaving more crystalline regions and increasing the CI value. 

### 3.8. Thermogravimetric Analysis (TGA)

**Figure 5:**
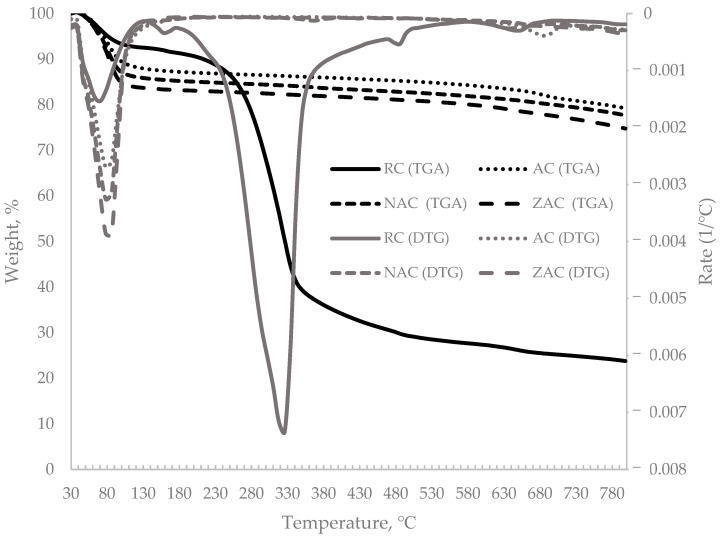
The TG and DTG curves for all types of cassava stem adsorbents.

The thermal stability for all types of cassava stem adsorbent was investigated through thermogravimetric analysis. The weight loss curve (TG) and the derivative thermogravimetric curve (DTG) are illustrated in Figure 5. All samples experienced initial weight loss through 100 °C, due to the evaporation of moisture and volatile materials. Significant weight loss only happened to the raw sample, with material loss at as much as 61.7% observed at in temperature range 180 to 472 °C due to the dehydration of polymer chains of cellulose and hemicellulose. At the end of the heating process, the raw cassava stem recorded the least residue amount, 25.29%. For all samples, minor weight loss was recorded in the temperature range 650 to 700 °C due to the opening of new pores through cell wall deterioration [65]. The activated cassava stem AC showed 79.31% residual weight, NAC at 77.77% and ZAC at 75.74%. The lower percentage of residual weight by the surface-modified activated carbon could be due to the degradation of the remaining surface modification chemical on the material’s surface.

### 3.9. Batch Adsorption Studies of Ofloxacin Adsorption

#### 3.9.1. The Effect of pH

**Figure 6:**
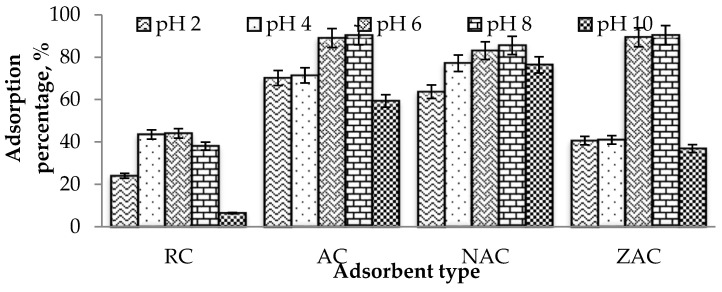
Plot of the adsorption percentage of ofloxacin onto all types of cassava stem adsorbents against different initial pH of the adsorbate (conditions: contact time = 120 min; initial ofloxacin concentration = 100 ppm; temperature = 35 °C; dosage = 1.5 g/L).

The effects of pH on the ofloxacin adsorption onto all types of cassava stem adsorbents are shown in Figure 6. All samples recorded the highest percentage of ofloxacin adsorption at pH 8, followed by adsorption at pH 6. The ofloxacin exists in different species at different ranges of pH [66]. Ofloxacin exists as a zwitterionic compound at pH between 6 and 8. At pH < 6 and pH > 8, the ofloxacin would exist as positively and negatively charged species, respectively. Therefore, considerable ofloxacin removal happened at pH 6 and 8 due to the high electrostatic interactions between the cassava stem adsorbents and ofloxacin molecules, attributable to different charge molecules at this pH. When too low, the pH value contributed to decreased ofloxacin adsorption percentage due to the electrostatic repulsion between the positively charged ofloxacin. It positively charged the surrounding, while at higher pH, the repulsion between the adsorbent - adsorbate occurred due to the negatively charged adsorbent surface. 

#### 3.9.2. The Effect of Contact Time

**Figure 7:**
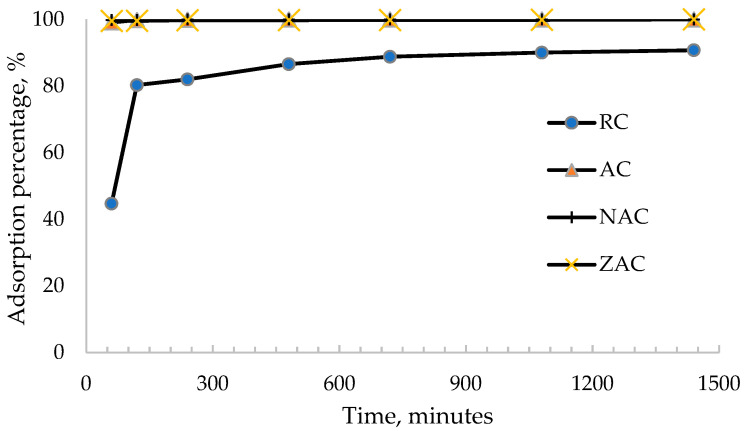
The plot of the adsorption percentage of ofloxacin onto all types of cassava stem adsorbents against different contact times (pH 5; initial ofloxacin concentration = 100 PPM; temperature = 35 °C; adsorbent dosage = 1.5 g/L).

The effect of contact time against the adsorption percentage of ofloxacin for all types of cassava stem adsorbents is displayed in Figure 7. Based on this figure, the highest percentage of ofloxacin adsorption was obtained at 180 min by ZAC (89.92%), followed by the activated cassava stem (89.62%), NAC (86.43%) and the raw cassava stem (55.19%). The ZAC achieves equilibrium after 75 min, the activated cassava stem at 90 min, the NAC at 120 min and the raw cassava stem at 180 min. The nonporous structure of the ZAC samples resulted in rapid adsorption of ofloxacin onto the external surface of these samples, along with a short diffusion path of ofloxacin in the shallow pore area [67], despite their insignificantly low removal percentage as compared to the other surface-modified adsorbents.

#### 3.9.3. The Effect of Temperature and Initial Concentration of Adsorbate

**Figure 8:**
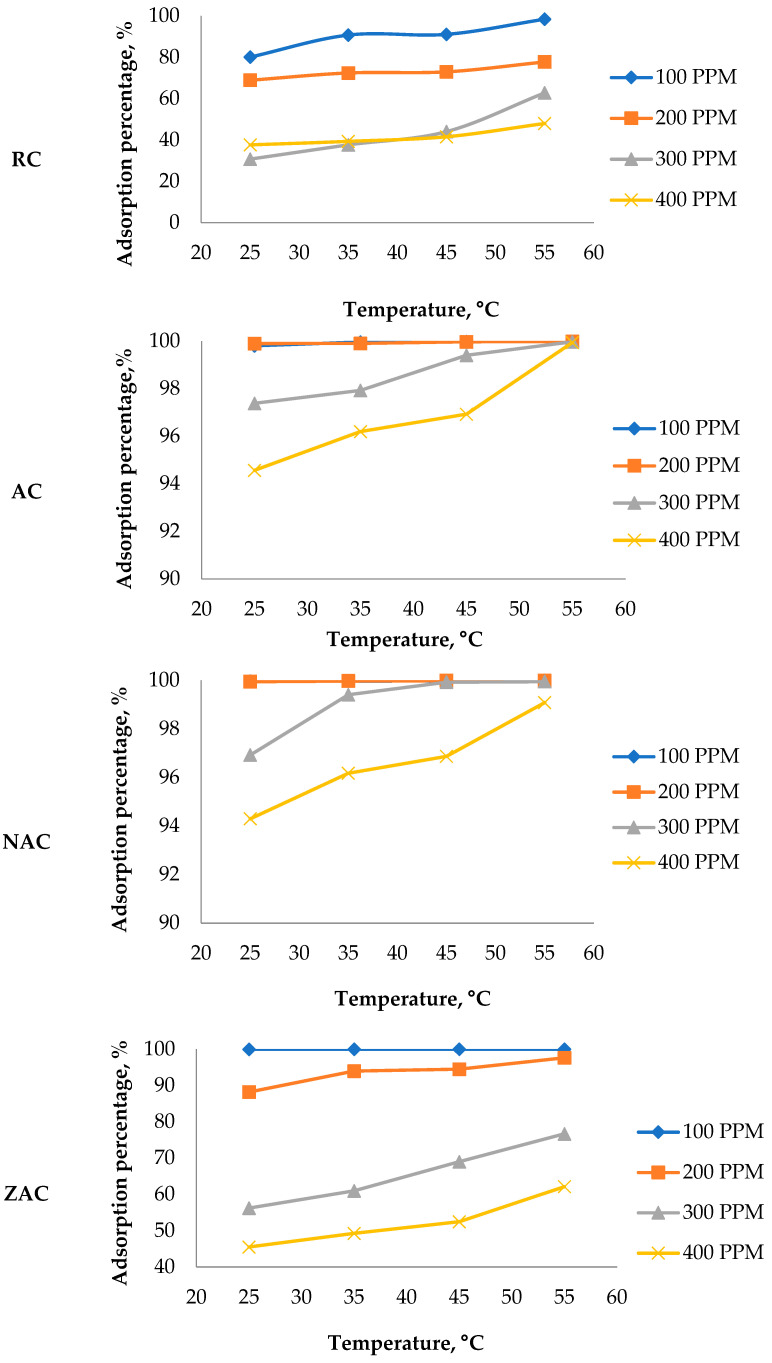
Plot of the ofloxacin adsorption percentage against temperature as a function of the initial concentration of the ofloxacin solution for different cassava stem adsorbents (condition: pH 5; contact time = 180 min; adsorbent dosage = 1.5 g/L).

Figure 8 shows that the percentage of ofloxacin adsorption increased as the temperature rose. It indicates that the adsorption of ofloxacin onto the surface of cassava stem adsorbents was an endothermic process [68]. Most samples recorded a high percentage of ofloxacin adsorption at an initial concentration of 100 ppm and a temperature of 55 °C, excluding the raw cassava stem. ZAC showed 94.44% adsorption, the activated cassava stem at 93.89%, NAC at 91.98% and the raw cassava stem at 59.10% adsorption. The adsorbents also showed good removal of ofloxacin up to the initial concentration of 100 ppm at a temperature of 55 °C. The ofloxacin removal percentage of cassava stem adsorbents was considered high and adequate in wastewater containing ofloxacin, which commonly contains much less than 50 ppm [11]. 

#### 3.9.4. The Effect of Adsorbent Dosage

**Figure 9:**
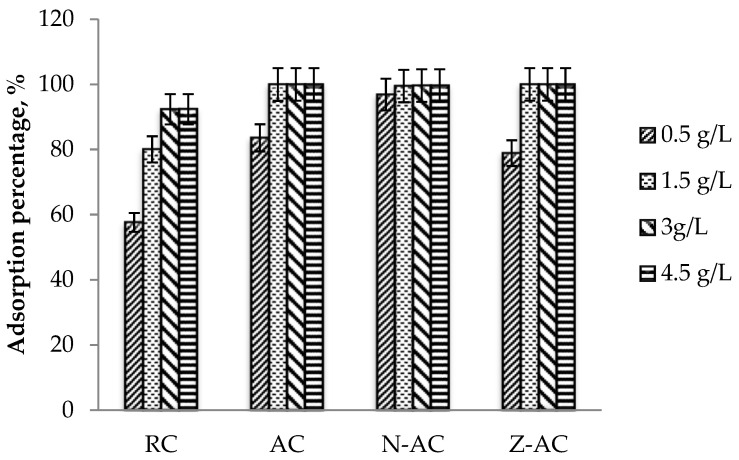
Plot of the adsorption percentage of ofloxacin onto all types of cassava stem adsorbents against different adsorbent dosages (condition: pH 5; contact time = 180 min; initial ofloxacin concentration = 100 ppm; temperature = 55 °C).

The effect of adsorbent dosage on the removal of ofloxacin is displayed in Figure 9. The adsorbent dosage of 0.5 g/L for NAC was sufficient to adsorb the 100 ppm ofloxacin concentration at 96.85% removal. For ZAC, the adsorption was 78.89% using the same amount of adsorbent dosage. At adsorbent dosage above 1.5 g/L, both NAC and ZAC showed ofloxacin uptake of over 99%. The simple explanation is that as the adsorbent dosage was increased, the availability of the free adsorption sites on the surface of the carbon was also increased, resulting in a higher adsorption percentage. Therefore, 1.5 g/L is the optimum adsorbent dosage as a higher dosage will not significantly improve the ofloxacin removal percentage, besides causing material waste. Meanwhile, the AC and RC showed 83.59% and 57.63% ofloxacin removal at a 0.5 g/L adsorbent dosage. This result proves the improvement of adsorption capacity after the activation process and surface modification of the activated carbon.

### 3.10. Thermodynamic Study of Ofloxacin Adsorption

**Figure 10:**
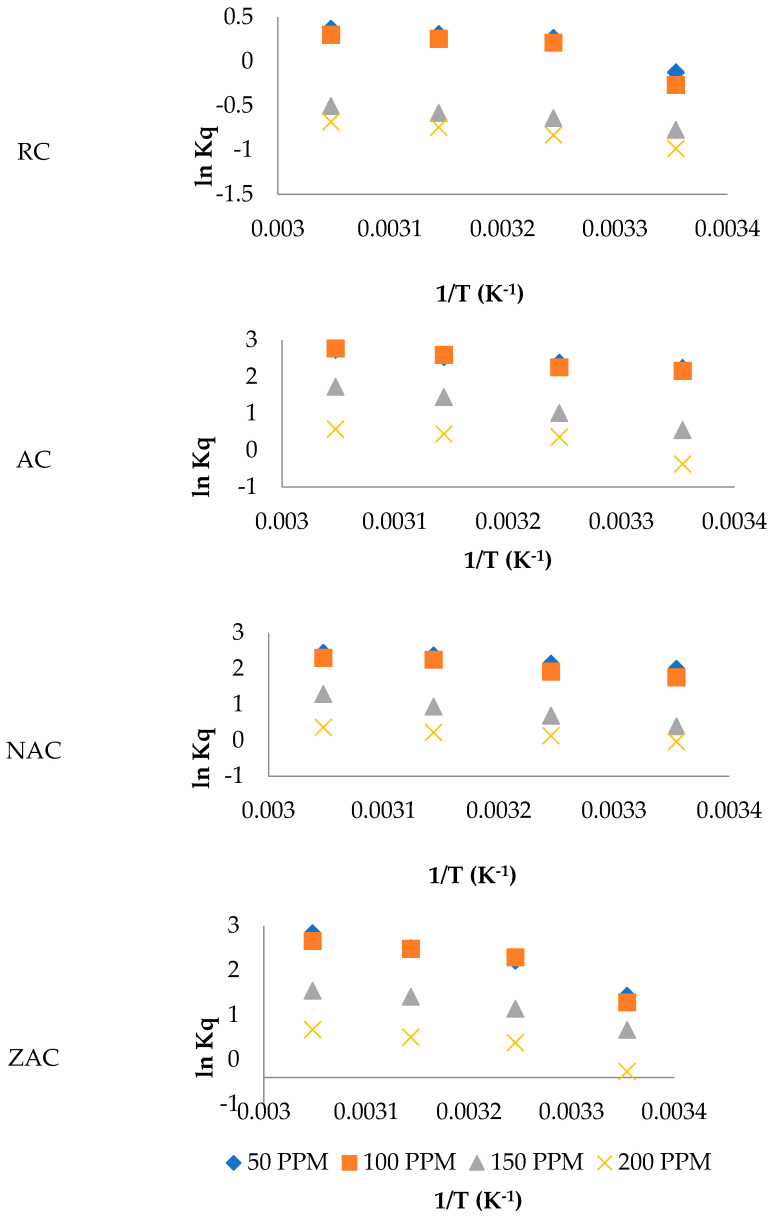
The plots of ln Kq against 1/T as a function of initial concentrations of ofloxacin.

The study on the thermodynamics of ofloxacin adsorption onto the cassava stem adsorbents was used to understand further the adsorption mechanism between the ofloxacin molecule and the surface of the cassava stem adsorbents. The plots of ln Kq against 1/T as a function of initial concentrations of ofloxacin are shown in Figure 10. The linear equations and R-squared values were generated from each of these plots. The linear equations generated: Gibbs free energy change (ΔG°), enthalpy change (ΔH°) and entropy change (ΔS°), are presented in Table 8.

The negative values of ΔG° were detected at all initial concentrations of ofloxacin and all set temperatures. It revealed that the adsorption of ofloxacin onto the surface of AC, NAC and ZAC was spontaneous at all investigated concentrations and temperatures. It was also found that these adsorbents had good ofloxacin adsorption efficiency even at low temperatures and high initial concentrations. The efficient removal ofloxacin was observed on RC, NAC and ZAC, showing a spontaneous process at almost all initial concentrations and temperatures except at an initial concentration of 200 ppm and a set temperature of 25 °C. Meanwhile, the lowest ofloxacin adsorption efficiency was observed for the raw cassava stem adsorbent. A nonspontaneous process was shown at all temperatures for high initial concentrations of ofloxacin (150 and 200 ppm) and temperatures of 25 °C for initial concentrations of 50 and 100 ppm. 

The value of ΔG° could also be used to differentiate the mechanism of adsorbate adsorption onto the adsorbent either through physisorption or chemisorption. The adsorption was said to occur through physisorption if the value of ΔG° is in the range −20 to 0 kJ/mol, while the chemisorption was said to happen if the value of ΔG° is in the range −400 to −80 kJ/mol [69]. The value of ΔG° for all prepared adsorbents in this study showed that the adsorption of ofloxacin onto the surface of cassava stem adsorbents occurred by a physical adsorption mechanism.

The positive value of ΔH° that was shown by all types of cassava stem adsorbents revealed that the adsorption of ofloxacin onto the cassava stem adsorbents was endothermic. Meanwhile, the positive values of ΔS° on all types of cassava stem adsorbents revealed that the movement of the adsorbed ofloxacin on the cassava stem adsorbents was not restricted as compared to the movement of ofloxacin in solution [66].

### 3.11. Kinetic Studies of Ofloxacin Adsorption

**Figure 11:**
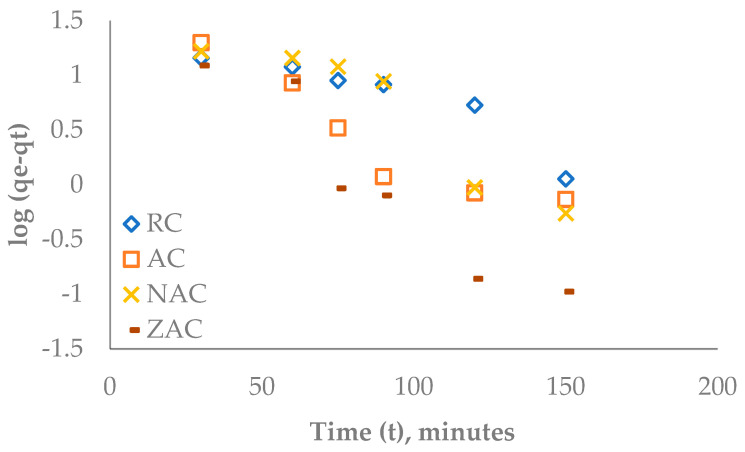
Plot of log (qe − qt) versus time t for the pseudo-first-order model for the adsorption of ofloxacin onto all types of cassava stem adsorbents.

The pseudo-first-order kinetic model in the plot of log (qe − qt) versus time for all types of cassava stem adsorbents is displayed in Figure 11. These plots give the linear equation and R-squared value presented in Table 9. The intercept and slope of the linear equation were used to calculate the parameters k_1_ and qe, where the k_1_ and qe represent the pseudo-first-order rate constant and the amount of ofloxacin adsorbed onto the surface of the cassava stem adsorbents at equilibrium, respectively. The R-squared value for all types of cassava stem adsorbents was less close to 1, indicating low conformity to the pseudo-first-order kinetic model.

**Figure 12:**
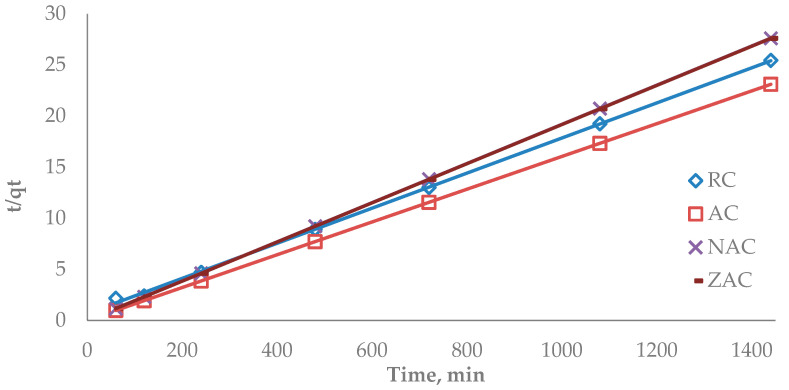
Plot of t/qt versus time t for the pseudo-second-order model for the adsorption of ofloxacin onto all types of cassava stem adsorbents.

The pseudo-second-order kinetic model was developed based on the theory that the rate-limiting step might be chemical adsorption, which involves valence forces by electron sharing between the adsorbate and adsorbent. A linear form of the pseudo-second-order kinetic model is t/qt = 1/h + (1/q_e_) · t, where h is the initial sorption rate, as qt/t = 0 [70]. Figure 12 shows the plots of t/qt against time. The slope and intercept were obtained from the plot’s linear equations and were used to calculate parameters k_2_ and qe, respectively. The parameter k_2_ indicates the pseudo-second-order rate constant, and qe indicates the adsorption capacity at equilibrium. The parameter h, which specifies the initial adsorption rate, was calculated from h = k_2_qe^2^, presented in Table 9. The h value followed the order: ZAC > AC > NAC > RC. This showed that the ZAC had the highest initial rate of ofloxacin adsorption, followed by the other samples. All types of cassava stem adsorbents showed correlation coefficient values close to 1, indicating ofloxacin adsorption onto the surface of all prepared cassava stem adsorbents fitted well with the pseudo-second-order kinetic model. As calculated from the pseudo-second-order equation, the maximum adsorption values were 42.37, 62.11, 62.89 and 58.82 mg/g for RC, AC, NAC and ZAC, respectively. 

Table 10 shows the maximum adsorption capacity between different adsorbents used for ofloxacin uptake from water. There is still limited research on the ofloxacin adsorption. The data are more limited when finding the maximum adsorption capacity based on the kinetic studies results. Based on the collected literature, the cassava-stem-based activated carbon possessed average adsorption capacity among the others. This is good enough, considering the low raw material cost for activated carbon production. 

## 4. Conclusions

Surface-modified activated carbon from cassava stem was produced in this work. NaOH and ZnCl_2_ were used as a surface modifier of the activated carbon. The fixed carbon content was higher for AC when compared to RC. The surface area analysis found that the activated carbon treatment with NaOH and ZnCl_2_ increases the surface area due to the removal of organic content by the chemicals. Better ofloxacin adsorption for all activated carbon samples was recorded at pH 8 due to the high electrostatic interactions between the cassava stem adsorbents at this pH. All activated carbon samples achieved maximum adsorption at 180 min contact time. Higher solution temperatures result in higher ofloxacin adsorption. Highest adsorption was shown at 55 °C. It indicates an endothermic reaction, supported by the positive value of ΔH° in the thermodynamic studies. At adsorbent dosage above 1.5 g/L, both NAC and ZAC showed ofloxacin uptake greater than 99%. The negative values of ΔG° revealed that the adsorption of ofloxacin onto the surface of AC, NAC and ZAC was spontaneous. The higher R^2^ values indicate that the adsorption process follows the pseudo-second-order equation of kinetic study. The maximum adsorption capacities are 42.37, 62.11, 62.89 and 58.82 mg/g for RC, AC, NAC and ZAC. Therefore, NAC has the highest adsorption capacity among the others. The adsorption capacity is above average compared to previous work by other researchers. While this work focused on ofloxacin adsorption, these activated carbons are also a possible material for the pharmaceutical industry’s wastewater treatment, and for the removal of dye, heavy metals and other water contaminants.

## Figures and Tables

**Table 1:** List of researched adsorbents for ofloxacin adsorption from water.

No.	Adsorbent	Reference
1	Calcined Verde-Lodo Bentonite Clay	[6]
2	Chitosan/Biochar Composite	[7]
3	Boron Nitride Nanosheets	[8]
4	Zeolite	[9]
5	Deep Eutectic Solvent (Choline Chloride-Based) Functionalised Rice Husk Ash	[10]
6	The Organic Waste-Derived Biochar	[11]
7	Rgo-Mos2 Heterostructure	[12]
8	Modified Thermally Activated Kaolin	[13]
9	Magnetic Zeolites	[14]
10	Cotton Stalk Biochar	[15]

**Table 2:** Parameters for batch adsorption studies.

Parameter	Values
Contact time, min	60, 120, 240, 480, 720, 1080 and 1440
pH	3, 5, 7, 9 and 12
Temperature, °C	25, 35, 45 and 55
The initial concentration of adsorbate	100, 200, 300 and 400 ppm
Adsorbent dosage	0.5, 1.5, 3.0 and 4.5 g/L

**Table 3:** Proximate analysis of surface-modified and nonsurface-modified activated-carbon-based cassava stem.

Samples	Moisture Content %	Volatile Content %	Ash Content %	Fixed Carbon Content %
RC	64.75 ± 0.70	84.80 ± 0.47	2.94 ± 0.12	12.26 ± 0.42
AC	6.83 ± 0.07	47.07 ± 0.59	3.42 ± 0.31	49.51 ± 0.65
NAC	6.57 ± 0.20	49.17 ± 0.46	5.00 ± 0.34	45.83 ± 0.47
ZAC	6.39 ± 0.36	48.44 ± 0.54	5.77 ± 0.34	45.79 ± 0.87

RC = raw cassava stem, AC = activated cassava stem, NAC = sodium hydroxide surface-modified cassava actived carbon, ZAC = zinc chloride surface-modified cassava activated carbon.

**Table 4:** Surface areas and pore size for all types of cassava stem adsorbents.

Types of Cassava Stem Adsorbents	S_BET_ (m^2^/g)	Average Pore Size (nm)	S_mic_ (m^2^/g)	S_mes_ (m^2^/g)	Pore Volume (cm^3^/g)		
					Total Pore Volume	V_mic_	V_mes_
RC	0.765	-	0.705	0.060	0.00040	0.00023	0.00017
AC	674.402	1.879	594.557	79.845	0.29663	0.24339	0.05325
NAC	847.725	1.990	685.202	162.523	0.38125	0.28240	0.09886
ZAC	712.184	1.951	580.666	131.518	0.31466	0.23847	0.07619

**Table 5:** The EDX findings for the surface of cassava stem adsorbents.

Element (Wt%)	RC	AC	NAC	ZAC
O	72.50	70.97	71.87	70.90
C	27.19	26.26	26.74	26.36
Ca	_	0.66	0.50	0.37
Mg	_	0.70	0.43	_
K	0.31	1.41	0.11	_
Na	_	_	0.33	_
Cl	_	_	_	0.20
Zn	_	_	_	2.18
Totals	100.00	100.00	100.00	100.00

**Table 6:** The C, O, H, N and S elements that are present on the surface of representative cassava stem adsorbents.

Element (Wt%)	RC	AC	NAC	ZAC
C	40.48	67.00	61.31	58.99
O	52.98	30.29	35.47	38.09
H	5.40	1.82	2.24	2.04
N	1.07	0.84	0.91	0.80
S	0.067	0.055	0.072	0.079

**Table 7:** The pHzpc value for all types of cassava stem adsorbents.

Samples	pHzpc Value
RC	6.53
AC	9.20
NAC	9.20
ZAC	7.10

**Table 8:** Thermodynamic parameters for the adsorption of ofloxacin onto all types of cassava stem adsorbents.

Sample	Initial Adsorbate Concentration	Linear Equation	R-Squared Value	∆H° (kJ/mol)	∆S° (kJ/mol/K)	∆G° (kJ/mol)			
						298.15 K	308.15 K	318.15 K	328.15 K
RC	50 ppm	y = −1503.7x + 5.0135	0.7950	12.5018	0.0417	0.0742	−0.3426	−0.7594	−1.1763
	100 ppm	y = −1725.9x + 5.6454	0.7516	14.3491	0.0469	0.3552	−0.1142	−0.5835	−1.0529
	150 ppm	y = −852.11x + 2.1022	0.9771	7.0844	0.0175	1.8735	1.6987	1.5239	1.3491
	200 ppm	y = −970.77x + 2.2955	0.9677	8.0710	0.0191	2.3809	2.1900	1.9992	1.8083
AC	50 ppm	y = −1586.9x + 7.5469	0.9883	13.1935	0.0627	−5.5139	−6.1414	−6.7688	−7.3963
	100 ppm	y = −2118.9x + 9.2244	0.9560	17.6165	0.0767	−5.2491	−6.0160	−6.7829	−7.5498
	150 ppm	y = −3914.8x + 13.704	0.9934	32.5477	0.1139	−1.4221	−2.5614	−3.7008	−4.8401
	200 ppm	y = −2895.1x + 9.5102	0.8082	24.0699	0.0791	0.4958	−0.2949	−1.0856	−1.8762
NAC	50 ppm	y = −1554x + 7.2028	0.9705	12.9199	0.0599	−4.9345	−5.5333	−6.1322	−6.7310
	100 ppm	y = −1924.34x + 8.2105	0.9428	15.9986	0.0683	−4.3537	−5.0363	−5.7190	−6.4016
	150 ppm	y = −2889.3x + 10.066	0.9922	24.0216	0.0837	−0.9302	−1.7670	−2.6039	−3.4408
	200 ppm	y = −1256.2x + 4.1858	0.9920	10.4441	0.0348	0.0682	−0.2798	−0.6278	−0.9758
ZAC	50 ppm	Y = −4403.4x + 16.325	0.9488	36.610	0.1357	−3.8569	−5.2141	−6.5714	−7.9286
	100 ppm	Y = −4275.5x + 15.852	0.8370	35.547	0.1318	−3.7477	−5.0657	−6.3836	−7.7015
	150 ppm	Y = −2859.4x + 10.333	0.9498	23.773	0.0859	−1.8406	−2.6997	−3.5588	−4.4178
	200 ppm	Y = −2906.9x + 9.6182	0.8719	24.168	0.0800	0.3262	−0.4735	−1.2731	−2.0728

RC = raw cassava stem, AC = activated cassava stem, NAC = sodium hydroxide surface-modified cassava activated carbon, ZAC = zinc chloride surface-modified cassava activated carbon.

**Table 9:** Pseudo-first-order and pseudo-second-order kinetic parameters for the adsorption of ofloxacin onto all types of cassava stem adsorbents.

	Pseudo First Order				Pseudo Second Order				
Sample	Linear Equation	R-Squared Value	q_e_ (mg/g)	k_1_ (min^−1^)	Linear Equation	r−Squared	q_e_ (mg/g)	k_2_ (g/mg/min)	h (mg/g/min)
RC	y = −0.0086x + 1.5654	0.8433	36.7621	0.0198	y = 0.0236x + 1.1242	0.9745	42.3729	0.0005	0.8895
AC	y = −0.0128x + 1.5523	0.8759	35.6697	0.0295	y = 0.0161x + 0.2723	0.9955	62.1118	0.0010	3.6724
NAC	y = −0.0142x + 1.9251	0.8660	84.1589	0.0327	y = 0.0159x + 0.4897	0.9834	62.8931	0.0005	2.0421
ZAC	y = −0.0193x + 1.7011	0.9059	50.2458	0.0444	y = 0.0170x + 0.1840	0.9960	58.8235	0.0016	5.4348

RC = raw cassava stem, AC = activated cassava stem, NAC = sodium hydroxide surface-modified cassava activated carbon, ZAC = zinc chloride surface-modified cassava activated carbon.

**Table 10:** Comparison of the maximum adsorption capacity for different ofloxacin adsorbents.

No.	Adsorbent	Adsorption Capacity, mg/g		Reference
		Langmuir	Pseudo-2nd Order	
1	Cotton Stalk Biochar	769.2	-	[15]
2	Calcined Verde-Lodo Bentonite Clay	160.81	-	[6]
3	Boron Nitride Nanosheets	72.50	-	[8]
4	Sodium Hydroxide-Modified Activated Cassava Stem	−	62.89	This work
5	Zinc Chloride-Modified Activated Cassava Stem	−	58.82	This work
6	Organic Waste-Derived Biochar	57.10	−	[11]
7	Modified, Thermally Activated Kaolin	45.275	−	[13]
8	Raw Cassava Stem	−	42.37	This work
9	Rgo-Mos2 Heterostructure	37.31	−	[12]
10	Deep Eutectic Solvent (Choline Chloride Based) Functionalised Rice Husk Ash	35.769	15.80	[10]
11	Magnetic Zeolites	11.6	−	[14]
12	Chitosan/Biochar Composite	6.64	3.06	[7]

## Data Availability

Data are available upon request from the corresponding author.
